# Improved diagnostic performance of plain radiography for cervical ossification of the posterior longitudinal ligament using deep learning

**DOI:** 10.1371/journal.pone.0267643

**Published:** 2022-04-27

**Authors:** Hee-Dong Chae, Sung Hwan Hong, Hyun Jung Yeoh, Yeo Ryang Kang, Su Min Lee, Minyoung Kim, Seok Young Koh, Yongeun Lee, Moo Sung Park, Ja-Young Choi, Hye Jin Yoo

**Affiliations:** 1 Department of Radiology, Seoul National University Hospital, Seoul, Republic of Korea; 2 Department of Radiology, Seoul National University College of Medicine, Seoul, Republic of Korea; 3 Institute of Radiation Medicine, Seoul National University Medical Research Center, Seoul, Republic of Korea; 4 Deepnoid Inc., Seoul, Republic of Korea; University of California San Francisco, UNITED STATES

## Abstract

**Background:**

A high false-negative rate has been reported for the diagnosis of ossification of the posterior longitudinal ligament (OPLL) using plain radiography. We investigated whether deep learning (DL) can improve the diagnostic performance of radiologists for cervical OPLL using plain radiographs.

**Materials and methods:**

The training set consisted of 915 radiographs from 207 patients diagnosed with OPLL. For the test set, we used 200 lateral cervical radiographs from 100 patients with cervical OPLL and 100 patients without OPLL. An observer performance study was conducted over two reading sessions. In the first session, we compared the diagnostic performance of the DL-model and the six observers. The diagnostic performance was evaluated using the area under the receiver operating characteristic curve (AUC) at the vertebra and patient level. The sensitivity and specificity of the DL model and average observers were calculated in per-patient analysis. Subgroup analysis was performed according to the morphologic classification of OPLL. In the second session, observers evaluated the radiographs by referring to the results of the DL-model.

**Results:**

In the vertebra-level analysis, the DL-model showed an AUC of 0.854, which was higher than the average AUC of observers (0.826), but the difference was not significant (*p* = 0.292). In the patient-level analysis, the performance of the DL-model had an AUC of 0.851, and the average AUC of observers was 0.841 (*p* = 0.739). The patient-level sensitivity and specificity were 91% and 69% in the DL model, and 83% and 68% for the average observers, respectively. Both the DL-model and observers showed decreases in overall performance in the segmental and circumscribed types. With knowledge of the results of the DL-model, the average AUC of observers increased to 0.893 (*p* = 0.001) at the vertebra level and 0.911 (*p* < 0.001) at the patient level. In the subgroup analysis, the improvement was largest in segmental-type (AUC difference 0.087; *p* = 0.002).

**Conclusions:**

The DL-based OPLL detection model can significantly improve the diagnostic performance of radiologists on cervical radiographs.

## Introduction

Ossification of the posterior longitudinal ligament (OPLL) of the cervical spine is one of the most common causes of cervical myelopathy in Eastern Asia, including Korea and Japan [1]. OPLL results in the replacement of ligamentous tissue by the abnormal ectopic bone formation and can lead to persistent compression of the spinal cord. As a result, various degrees of neurologic deficits and spastic cervical myelopathy symptoms can lead to the restriction of activities of daily living and low quality of life [2].

Cervical radiography is usually performed as an initial diagnostic test for the assessment of symptoms from OPLL. On the conventional lateral radiography, OPLL appears as continuous or segmental ossifications posterior to the vertebral bodies along the course of the posterior longitudinal ligament (PLL). However, many instances of cervical OPLL can be overlooked on lateral plain radiographs due to the complex anatomic structures around the cervical spine and superimposition of the ossified ligaments with facet joints and osteophytes [3]. The overall false-negative rate of cervical OPLL in lateral radiographs was reported to be approximately 48% [4], and the interobserver variability showed only fair agreement in the lateral radiograph-based classification of cervical OPLL [5]. Computed tomography (CT) can most accurately delineate OPLL and is the diagnostic modality of choice for preoperative planning. CT can detect small lesions that are indistinct on simple radiographs and precisely assess the thickness and length of the OPLL. However, CT is not routinely performed as an initial evaluation in daily practice, and there is a limitation to its repeated use as a follow-up test due to the high radiation dose. Therefore, even in the era of cross-sectional imaging, plain radiography plays a fundamental role in the diagnosis and assessment of cervical OPLL.

Recent advances in artificial intelligence have shown promising results in various fields of radiology for the detection and characterization of lesions [6, 7]. In a recent study by Miura et al., they showed that deep learning can differentiate between cervical spondylosis and OPLL using lateral cervical radiographs with equal or superior diagnostic performance to that of spine surgeons [8]. However, they did not explore whether deep learning could improve observer performance when used as a secondary reader. Therefore, the purpose of this study was to investigate whether the DL model can improve the diagnostic performance of radiologists for cervical OPLL using plain radiographs.

## Materials and methods

The institutional review board of Seoul National University Hospital (IRB No. 1711-133-901) approved this retrospective study with a waiver of informed consent.

### Patients

**Fig 1:**
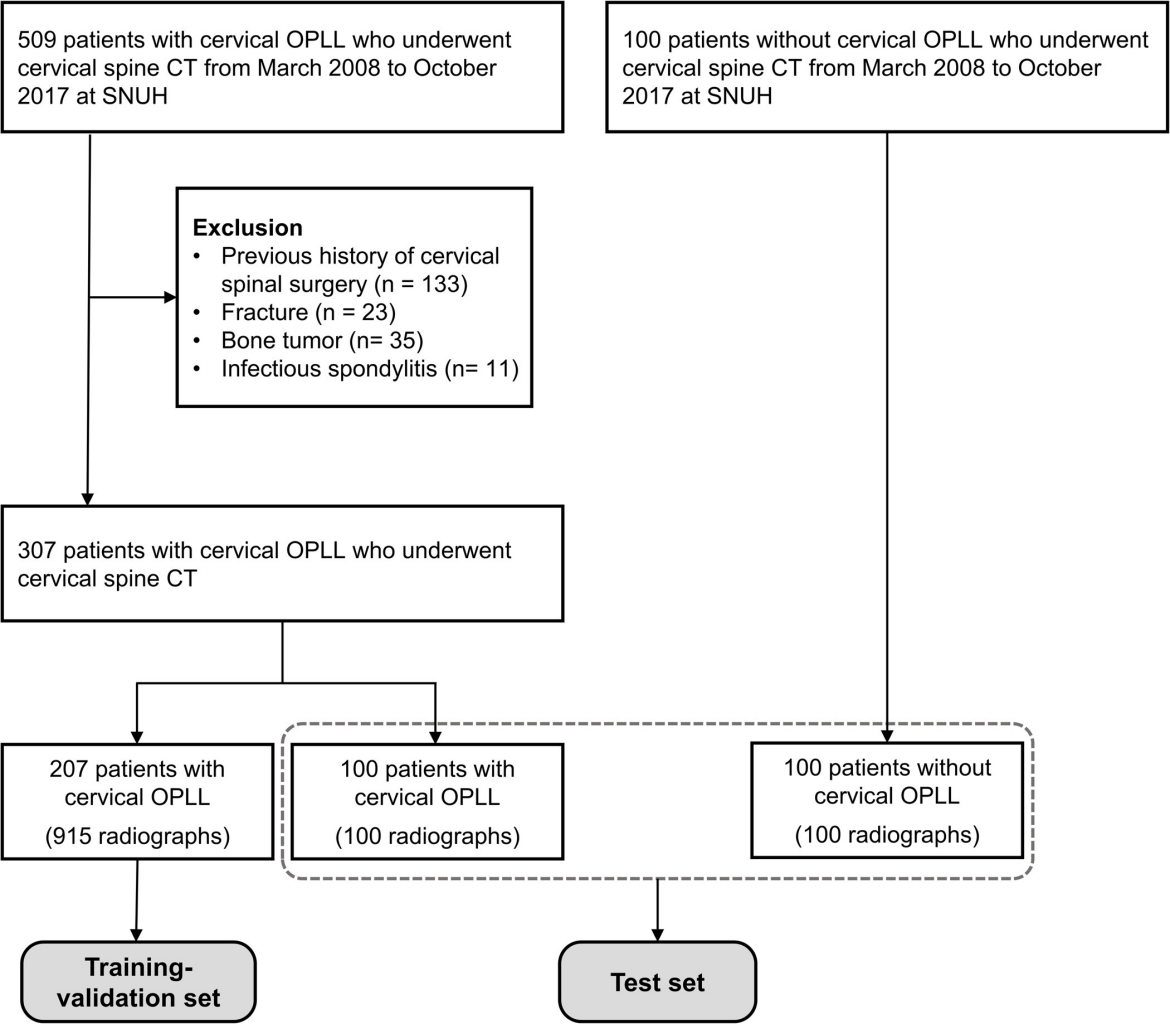
Flow diagram of data inclusion and allocation.

We retrospectively collected 5289 patients who underwent cervical spine CT between March 2008 and October 2017. Of these, 509 patients were diagnosed with cervical OPLL. We excluded patients with a previous history of spinal surgery (n = 133), fracture (n = 23), tumors (n = 35), or infectious spondylitis (n = 11) involving the cervical spine. Next, we split the remaining 307 patients with OPLL into a training-validation (n = 207) and test set (n = 100). For the test set, we additionally collected a convenient sample of 100 patients without OPLL (Fig 1). Finally, 915 radiographs from 207 patients with OPLL (157 men; mean age 59.7 ± 9.9 years; range 30–84 years) were used for the training-validation set, and 100 lateral cervical radiographs from 100 patients with cervical OPLL (68 men; mean age 59.4 ± 11.3 years; range 32–81 years) and 100 radiographs from 100 patients without OPLL (54 men; mean age 51.1 ± 16.6 years; range 19–83 years) were used for the test set (Table 1). Multiple radiographs were used from the same patient for the training-validation set to increase the training data, but only one image per patient was used for the test set. The patients in the training-validation and test sets were exclusive to each of the other datasets. The presence or absence of OPLL of the patients in the training-validation and test sets was confirmed on cervical spine CT, and we considered an ossification with more than 2 mm thickness measured in the axial plane as positive based on the previous study [9]. When multiple CT scans were available, we referred the CT scan closest to the study date of the cervical radiograph. For the test set, we selected a cervical radiograph performed on the day closest to the preoperative CT. The mean intervals between CT and radiographs were 205 days for training-validation set and 10 days for test set, respectively. All radiographs were obtained using DigitalDiagnost VM, DigitalDiagnost VR, DigitalDiagnost VH (Philips Healthcare, Best, The Netherlands), or DGR-C22M2A/KR (Samsung Healthcare, Seoul, Korea).

**Table 1:** Patient characteristics.

	Training-validation set	Test set		P value
		with OPLL	without OPLL	
**Number of patients**	207	100	100	
**Number of images**	915	100	100	
**Age, years**	59.7 ± 9.9	59.4 ± 11.3	51.1 ± 16.6	< 0.001
**Male sex, n (%)**	157 (75.8)	68 (68)	54 (54)	< 0.001
**OPLL subtype**				0.667
Continuous	38	16		
Segmental	122	62		
Mixed	30	17		
Circumscribed	17	5		

OPLL, ossification of the posterior longitudinal ligament.

### Data labeling and development of the DL model

A board-certified musculoskeletal radiologist (H.D.C.) with nine years of experience in spine imaging manually segmented OPLL along the boundary of the lesion on radiographs using commercial software (DeepPhi, Deepnoid, Seoul, Korea). During manual segmentation, the corresponding cervical CT images of the patient were referred to as ground truth, and the presence of OPLL lesion for each vertebral level was recorded. Then, according to the CT findings, the morphological types of OPLL were classified into four subtypes by the same radiologist using the classification system proposed by the Investigation Committee on OPLL of the Japanese Ministry of Public Health and Welfare [10]: continuous, segmental, mixed, and circumscribed-type.

**Fig 2:**
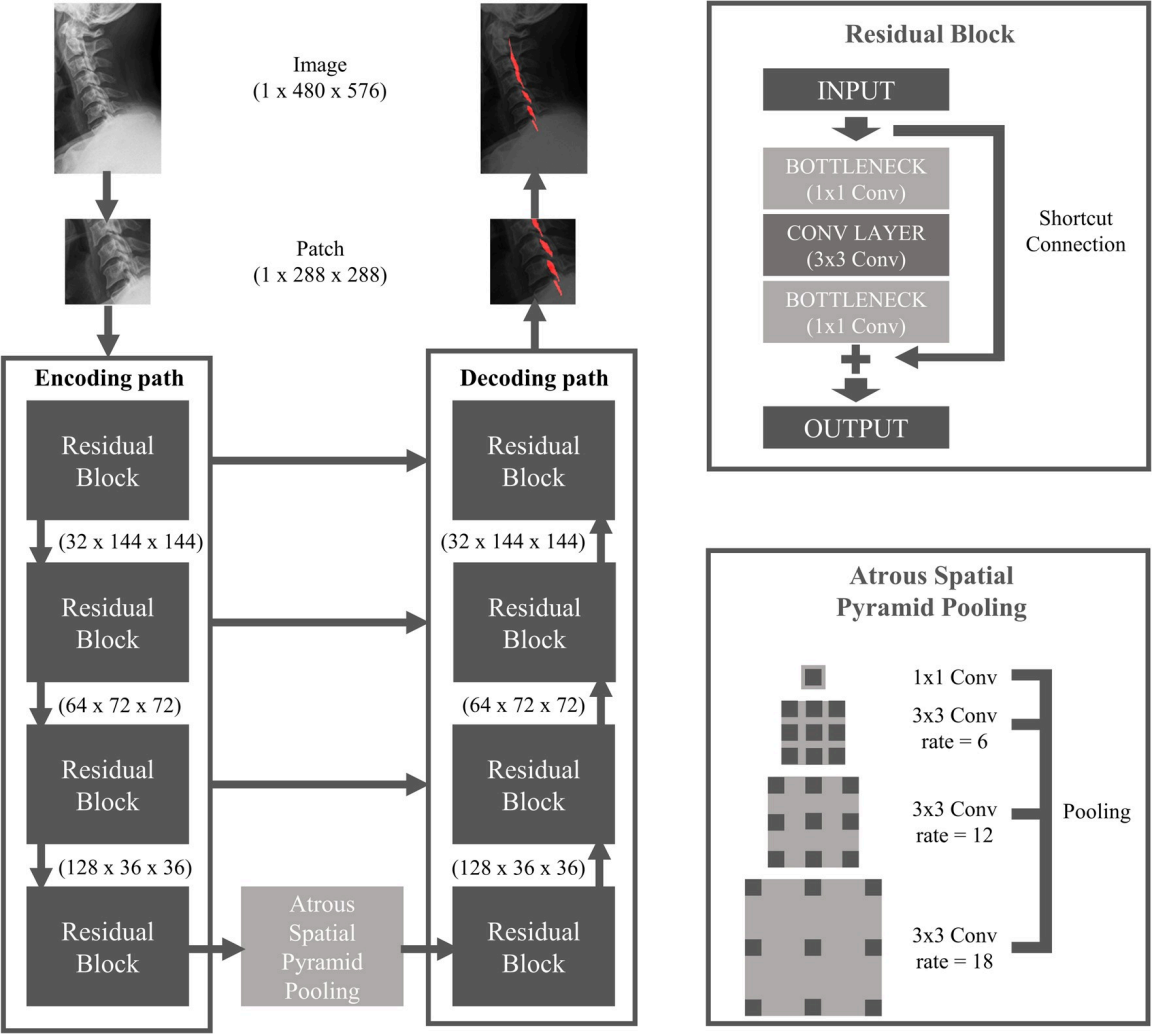
The architecture of the deep convolutional neural network used for OPLL segmentation. OPLL, ossification of the posterior longitudinal ligament.

Two-dimensional residual U-net with atrous spatial pyramid pooling was implemented and trained to segment cervical OPLL on lateral radiographs [11–13]. Cervical radiographs were resized to 480x576, and 288x288-sized patches were extracted from the image with a stride value of 32. Among the patches, those including at least one pixel of OPLL annotation were used for training. The initial filter size was 32, which was doubled after passing each convolutional layer. We used the Adam optimizer (β1 = 0.9, β2 = 0.999 and eps = 1x10^-8^) and Dice coefficient loss function. The initial learning rate was set to 0.0003 with a decay rate of 0.9 for every 2000 steps and the number of epochs was 300. The batch size of 5 and the dropout rate of 0.15 were used for the training. Segmentation results inferred at patch-level were recombined to reconstruct the entire image, and for the overlap between the patches, the results were determined by majority votes for multiple patches (Fig 2). We augmented our training data with several augmentation methods such as rotation, blurring, sharpening, brightness change, and the addition of Gaussian noise using the imgaug library (version 0.4.0) [14]. Our model was implemented with TensorFlow (version 2.4.1, https://www.tensorflow.org) [15] and the entire code can be found at https://github.com/moosungpark/opll.

### Evaluation of the diagnostic accuracy of DL model and radiologists

Per-vertebra and per-patient analysis was done to evaluate the diagnostic performance of the model. In per-vertebra analysis, the cervical spine was divided into six regions of interests (ROI) from C2 to C7, and for convenience, lesions located at the intervertebral disc level were considered to belong to the adjacent upper vertebral level. To assess the performance of the DL model, we recorded the lowest threshold at which OPLL lesions first appeared for each vertebral level, increasing the threshold value from 0.1 to 0.9 by an increment of 0.1, and that lowest threshold was used as the ROI-level probability of the corresponding vertebral level. For per-patient analysis, we used the highest probability among the six ROI-level probabilities as the patient-level probability score.

The observer performance test was conducted over two sessions, and in the first session, we compared the diagnostic performance of the DL model with those of observers. The second session was performed with an interval of 8 weeks, and each observer evaluated the image by referring to the results of the DL model. The observers consisted of three different subgroups by their level of experience in spine imaging: two radiology residents, two musculoskeletal radiology fellows, and two staff radiologists. A total of six observers independently reviewed the validation test data sets set on a commercial PACS program (INFINITT PACS, INFINITT Healthcare, Seoul, Korea). Diagnostic confidence in the presence of OPLL was scored at each vertebra-level ROI with a 5-point Likert scale: grade 1, definitely not present; grade 2, probably not present; grade 3, possibly present; grade 4, probably present; and grade 5, definitely present. As in the DL model, the highest confidence score among the six ROI was used as the patient-level confidence score.

### Statistical analysis

The Fisher’s exact test and one-way analysis of variance test were used to compare categorical and continuous variables between training and test sets. We used Cohen’s kappa with linear weights and the Fleiss multi-rater kappa to evaluate interobserver variability in the confidence scores in the diagnosis of OPLL among radiologists. Cohen’s κ values < 0.40 signified poor agreement, 0.41–0.60 moderate agreement, 0.61–0.80 good agreement, and ≥0.81 excellent agreement.

The observer performance test was analyzed using the R package RJafroc (version 2.0.1) [16]. Per-patient analysis was conducted using the area under the receiver operating characteristic (ROC) curve (AUC) analysis. The sensitivity and specificity of the DL model and average observers were calculated with the test set, and we selected the threshold at an activation value of 0.8 for the DL model and grade 3 for human observers, respectively. In the per-vertebra analysis, where there were multiple ROIs in one patient, the ROI-based ROC approach with the Obuchowski-Rockette-Hillis method [17] was applied to account for within-patient correlations of the clustered data. Regarding multiple-reader, multiple-case analysis, the diagnostic performance of the DL model was compared against individual observers using the fixed-reader, random-case (FRRC) method, and the random-reader, random-case (RRRC) method was used to compare the performance of the DL model to the average of the radiologists. The comparison of the AUC of individual observers between two sessions was compared using the FRRC method, and the RRRC method was used for the average AUC of observers. Finally, we performed subgroup analysis according to the vertebral level to evaluate the change in the diagnostic performance of the human observer and the DL model according to each vertebral level.

Statistical analyses were performed using R statistical software (ver. 4.0.5; R Foundation for Statistical Computing, Vienna, Austria). A *p*-value of < 0.05 was considered to indicate a significant difference.

## Results

### OPLL lesion characteristics

The results of interobserver variability for the confidence scores in the diagnosis of OPLL showed moderate agreement between two staff radiologists (κ = 0.56), good agreement between two radiology fellows (κ = 0.66), and poor agreement between two radiology residents (κ = 0.30) (S1 Table). The Fleiss κ for all raters was 0.28. When classified into subtypes according to OPLL morphology, there were 38 continuous-type (18%), 122 segmental-type (59%), 30 mixed-type (14%), and 17 circumscribed-type OPLL (8%) in the training set. For the test set, continuous-type was observed in 16 patients (16%), segmental-type in 62 patients (62%), mixed-type in 17 patients (17%), and circumscribed-type in 5 patients (5%).

### Comparison of diagnostic performances of the DL model and observers

In per-vertebra analysis (Table 2), the DL model showed an AUC of 0.854 (95% CI, 0.828–0.880), which was higher than the average AUC of 6 observers (0.826; 95% CI, 0.772–0.881), but the difference was not significant (*p* = 0.292). The fixed-reader, random-case analysis revealed that the performance of the DL model was higher than one fellow and resident (AUC, 0.854 vs. 0.788 and 0.754; *p* = 0.002 and < 0.001, respectively). And there was no significant difference in diagnostic performance between the DL model and two staff radiologists (*p* = 0.163 and 0.683, respectively). When subgroup analysis was performed according to the morphologic subtype of OPLL, both the DL model and observers showed the best performance in continuous-type (DL model: AUC, 0.897; average observers, AUC, 0.953) and the worst in circumscribed-type (DL model: AUC, 0.819; average observers, AUC, 0.684). Both the DL model and observers showed decreases in overall performance in the segmental and circumscribed-types compared to the continuous and mixed-types, and this decrease showed the tendency to be more prominent in human observers, especially in radiology residents. In the segmental-type, the AUC value of the DL model was significantly higher than those of the three observers (AUC, 0.825 vs. 0.707, 0.763, and 0.633; *P*-value range, < 0.001 to 0.027).

**Table 2:** Results of the observer performance test, including subgroup analysis, according to the morphologic subtype of OPLL.

	Per-vertebra analysis										Patient level	
	Total		Continuous		Segmental		Mixed		Circumscribed		Total	
	AUC (95% CI)	*p* value	AUC (95% CI)	*p* value	AUC (95% CI)	*p* value	AUC (95% CI)	*p* value	AUC (95% CI)	*p* value	AUC (95% CI)	*p* value
DL model	0.854 (0.828–0.880)	-	0.897 (0.839–0.956)	-	0.825 (0.786–0.864)	-	0.881 (0.845–0.916)	-	0.819 (0.637–1.001)	-	0.851 (0.799–0.903)	-
1st session (DL model vs. Observer)												
Staff 1	0.880 (0.846–0.914)	0.163	0.980 (0.961–0.999)	0.006	0.822 (0.772–0.872)	0.928	0.941 (0.888–0.995)	0.025	0.836 (0.751–0.920)	0.812	0.876 (0.832–0.920)	0.425
Staff 2	0.847 (0.812–0.881)	0.683	0.949 (0.905–0.993)	0.125	0.788 (0.744–0.833)	0.143	0.926 (0.882–0.970)	0.065	0.637 (0.367–0.907)	0.286	0.837 (0.788–0.886)	0.683
Fellow 1	0.868 (0.831–0.904)	0.481	0.975 (0.946–1.004)	0.009	0.807 (0.755–0.859)	0.552	0.926 (0.863–0.990)	0.145	0.763 (0.522–1.005)	0.605	0.878 (0.831–0.925)	0.407
Fellow 2	0.788 (0.745–0.832)	0.002	0.944 (0.898–0.990)	0.150	0.707 (0.655–0.758)	< 0.001	0.852 (0.764–0.940)	0.497	0.621 (0.374–0.867)	0.101	0.847 (0.795–0.898)	0.891
Resident 1	0.821 (0.782–0.859)	0.089	0.940 (0.913–0.966)	0.174	0.763 (0.712–0.814)	0.027	0.898 (0.853–0.943)	0.442	0.664 (0.328–1.001)	0.483	0.805 (0.745–0.864)	0.182
Resident 2	0.754 (0.707–0.801)	< 0.001	0.928 (0.871–0.986)	0.417	0.633 (0.581–0.685)	< 0.001	0.879 (0.817–0.942)	0.972	0.584 (0.349–0.819)	0.102	0.804 (0.744–0.863)	0.214
Average Observers^†^	0.826 (0.772–0.881)	0.292	0.953 (0.921–0.985)	0.069	0.753 (0.677–0.830)	0.073	0.904 (0.851–0.957)	0.369	0.684 (0.507–0.861)	0.291	0.841 (0.797–0.885)	0.739
2nd session (without DL model vs. with DL model)												
Staff 1	0.903 (0.872–0.933)	< 0.001	0.990 (0.981–1.000)	0.039	0.853 (0.808–0.899)	< 0.001	0.958 (0.909–1.006)	0.013	0.862 (0.787–0.938)	< 0.001	0.921 (0.887–0.955)	< 0.001
Staff 2	0.920 (0.896–0.944)	< 0.001	0.977 (0.955–0.998)	0.029	0.887 (0.854–0.920)	< 0.001	0.961 (0.917–1.004)	0.101	0.805 (0.607–1.004)	0.002	0.936 (0.906–0.965)	< 0.001
Fellow 1	0.912 (0.886–0.938)	0.002	0.979 (0.954–1.005)	0.253	0.861 (0.821–0.902)	0.012	0.977 (0.956–0.999)	0.091	0.789 (0.569–1.010)	0.082	0.936 (0.901–0.970)	0.005
Fellow 2	0.876 (0.843–0.910)	< 0.001	0.978 (0.951–1.005)	0.072	0.810 (0.759–0.860)	< 0.001	0.957 (0.916–0.997)	0.004	0.636 (0.372–0.901)	0.127	0.910 (0.873–0.948)	0.001
Resident 1	0.883 (0.854–0.913	< 0.001	0.975 (0.959–0.990)	< 0.001	0.843 (0.803–0.883)	< 0.001	0.945 (0.913–0.978)	< 0.001	0.715 (0.326–1.104)	0.485	0.877 (0.829–0.925)	0.002
Resident 2	0.861 (0.827–0.895)	< 0.001	0.975 (0.949–1.001)	0.028	0.786 (0.737–0.834)	< 0.001	0.932 (0.882–0.982)	< 0.001	0.761 (0.536–0.986)	0.025	0.884 (0.837–0.930)	0.006
Average Observers^§^	0.893 (0.862–0.924)	0.001	0.979 (0.962–0.997)	0.012	0.840 (0.795–0.885)	0.002	0.955 (0.920–0.990)	0.007	0.762 (0.582–0.941)	0.061	0.911 (0.876–0.945)	< 0.001

* *p* values and 95% CI are from the fixed-reader, random-case analysis comparing AUC between the DL model and individual observers.

^†^ *p* values and 95% CI are from the random-reader, random-case analysis comparing AUC between the DL model and average observers.

^‡^ *p* values and 95% CI are from the fixed-reader, random-case analysis comparing the AUC of individual radiologists between the 1st session (without DL model) and the 2nd session (with DL model).

^§^ *p* values and 95% CI are from the random-reader, random-case analysis comparing the AUC of individual radiologists between the 1st session (without DL model) and the 2nd session (with DL model).

DL, deep learning; AUC, area under the curve.

**Fig 3:**
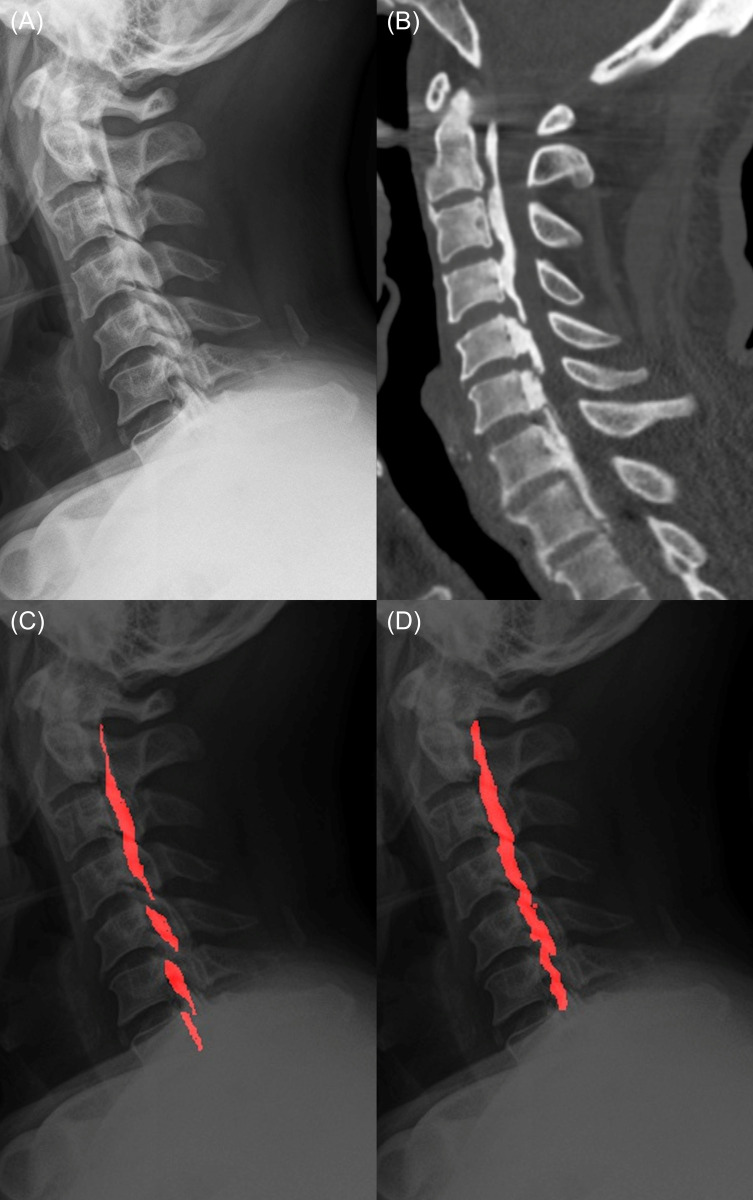
A 65-year-old male patient with mixed-type OPLL. (A) A lateral plain radiograph shows mixed-type OPLL along the posterior side of the vertebra. The lesion at the C7 level is obscured by the shoulder shadow. (b) In the sagittal image of cervical spine CT, ossifications ranging from the C2 to T1 level are clearly demonstrated (window width = 2000 HU, window level = 500 HU). (c) OPLL lesions annotated by a radiologist on the plain radiograph. (d) In the resulting image inferred by the deep-learning model, OPLL lesions at the C2-6 levels are well predicted, but the lesion located at the C7 level was not detected by the model. OPLL, ossification of the posterior longitudinal ligament.

**Fig 4:**
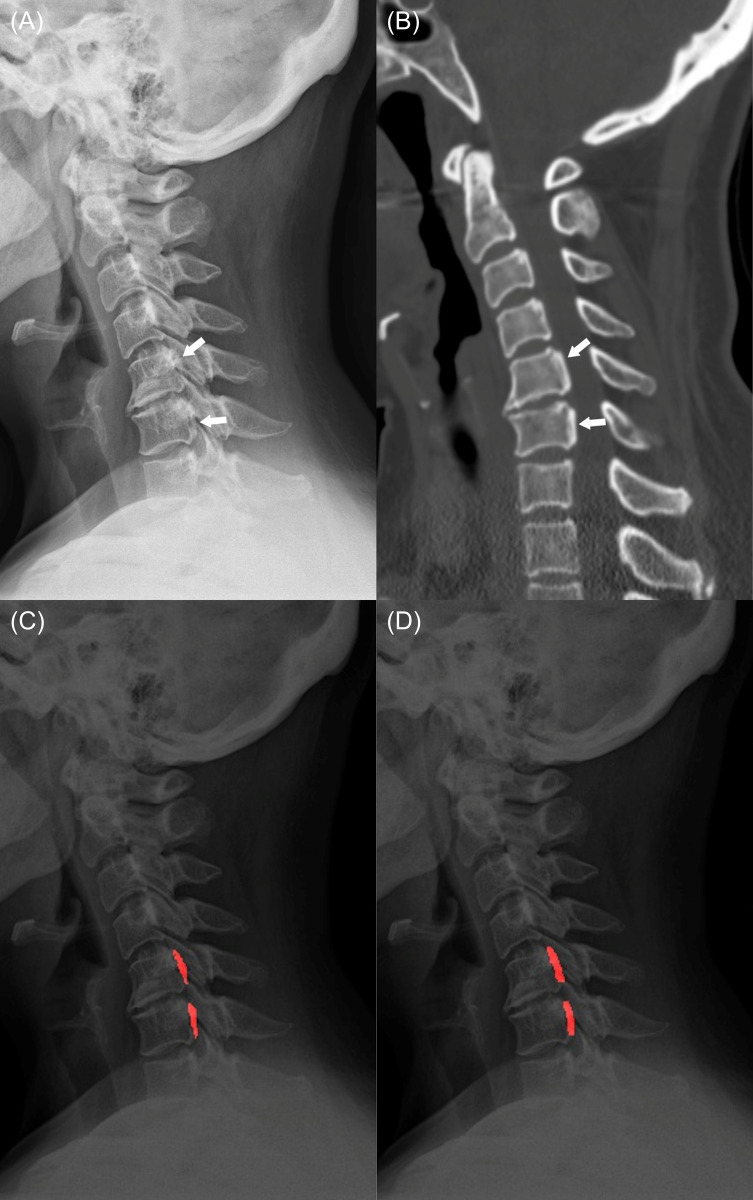
A 51-year-old female patient with segmental-type OPLL. (a) A lateral plain radiograph shows segmental-type OPLL (arrow) at the C5-6 level. (b) A sagittal image of cervical spine CT also demonstrates the segmental ossifications (arrow) at C5-6 level (window width = 2000 HU, window level = 500 HU). (c) OPLL lesions annotated by a radiologist on the plain radiograph. (d) The deep-learning model correctly predicted the segmental-type OPLL, which was overlooked by two observers. OPLL, ossification of the posterior longitudinal ligament.

In per-patient analysis, the performance of the DL model was AUC 0.851 (95% CI, 0.799–0.903) and that of average observers was AUC 0.841 (95% CI, 0.781–0.901) (*p* = 0.739). The patient-level sensitivity and specificity were 91% (95% CI, 83.6–95.8%) and 69.0% (95% CI, 59.0–77.9%) in the DL model, and 83.3% (95% CI, 80.1–86.2%) and 67.7% (95% CI, 63.8–71.4%) for the average observers, respectively. Representative cases are shown in Figs 3 and 4.

### Comparison of observer performances with and without the DL model

**Fig 5:**
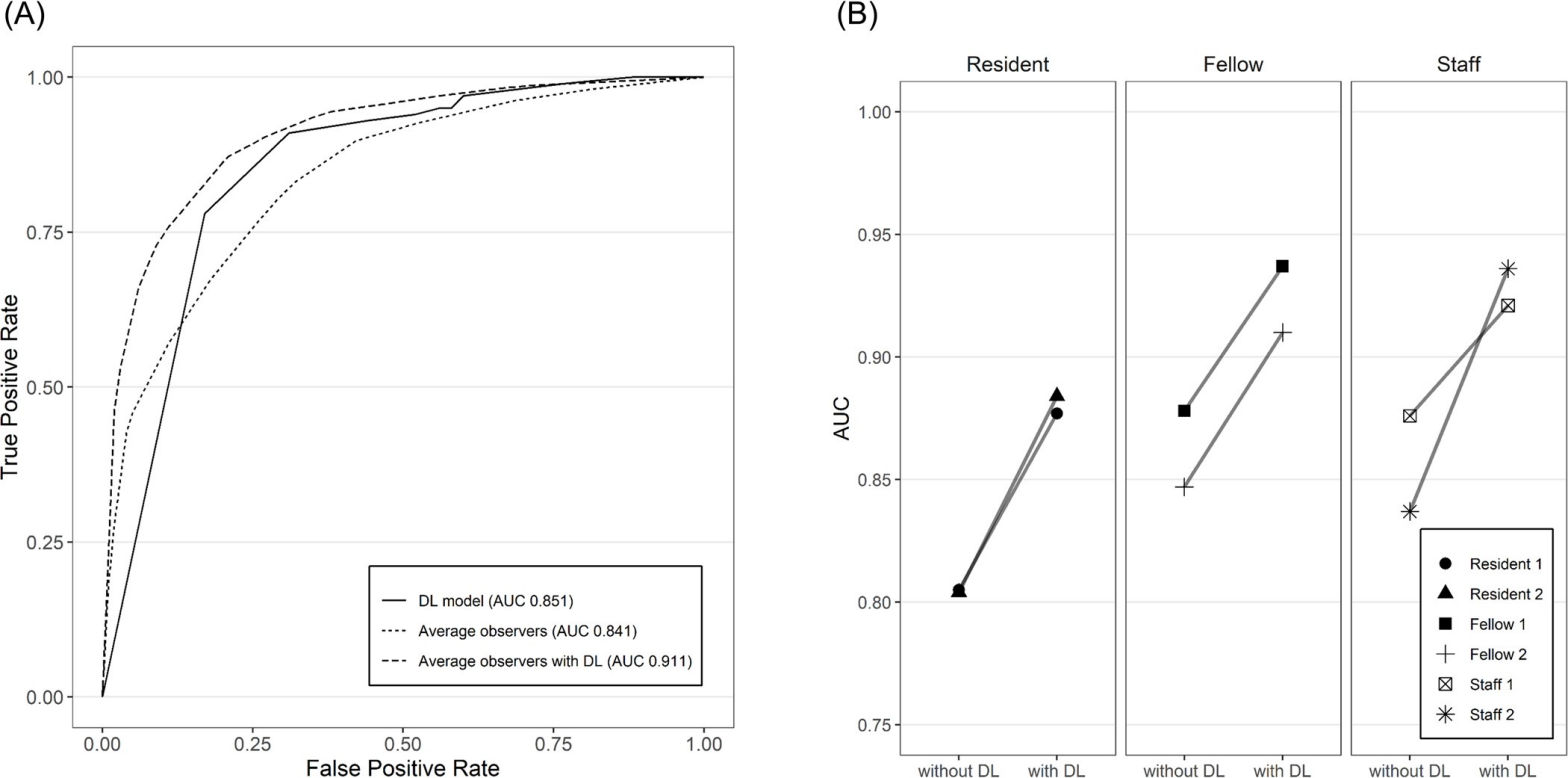
Comparison of observer performances with and without the DL model (a) ROC curve of the DL model and average observers in per-patient analysis. The AUC of the DL model alone was 0.851 (95% CI, 0.799–0.903), and that for average observers was 0.841 (95% CI, 0.781–0.901). The AUC of average observers improved to 0.911 (95% CI, 0.876–0.945) when referring to the results of the DL model. (b) Improved diagnostic performance of individual observers in per-patient analysis with the assistance of the DL model. ROC, receiver operating characteristics; DL, deep learning; AUC, area under the curve.

When referring to the result images of the DL model, the diagnostic performance of all observers was significantly improved regardless of their level of experience (Table 2). With knowledge of the DL model results, the average AUC of observers increased to 0.893 (*p* = 0.001) at per-vertebra analysis and 0.911 (*p* < 0.001) at per-patient analysis (Fig 5). When the performance of each observer group referring to the result of the DL model was compared with the DL model, the average AUC of residents, fellows, and staff radiologists were 0.872 (*p* = 0.320), 0.884 (*p* = 0.190), and 0.911 (*p* = 0.004), respectively, and staff radiologists showed significantly higher performance than the DL model. In the subgroup analysis according to the morphologic subtype, the improvement in diagnostic performance was largest in segmental-type (AUC difference 0.087; *p* = 0.002), and the increment was the smallest in continuous-type (AUC difference 0.026; *p* = 0.026).

### Subgroup analysis according to the vertebral level

The results of subgroup analysis at each vertebral level revealed that the diagnostic performance was best at the C2 level in both the DL model (AUC, 0.932) and average observers (AUC, 0.927) (Table 3). In both groups, performance tended to decrease toward the lower cervical level, and in particular, the DL model showed the lowest AUC value of 0.582 at the C7 level, which was significantly lower than that of the observers (AUC, 0.793) (*p* < 0.001).

**Table 3:** Subgroup analysis according to the vertebral level.

Vertebral level	DL model	Average Observers	*p* value*
C2	0.932 (0.862–1.001)	0.927 (0.902–0.951)	0.685
C3	0.904 (0.840–0.968)	0.904 (0.844–0.964)	0.996
C4	0.906 (0.862–0.949)	0.846 (0.782–0.911)	0.071
C5	0.865 (0.812–0.918)	0.829 (0.755–0.902)	0.319
C6	0.829 (0.765–0.893)	0.773 (0.667–0.878)	0.277
C7	0.582 (0.487–0.677)	0.793 (0.675–0.911)	< 0.001

Data are AUC and number in parentheses are 95% CI.

* *p* values and 95% CI are from the random-reader, random-case analysis comparing AUC between DL model and average observers.

DL, deep learning; AUC, area under the curve.

## Discussion

We developed a deep learning-based OPLL detection model and investigated its effect on improving diagnostic performance for OPLL using lateral cervical radiographs. The average AUC of observers increased from 0.826 to 0.893 (*p* = 0.001) at the per-vertebra analysis and from 0.841 to 0.911 (*p* < 0.001) at the per-patient analysis when referring to the results of the DL model. Both the DL model and radiologists showed higher diagnostic performance in the continuous and mixed-types than in the segmental and circumscribed-types. In addition, the improvement in diagnostic performance was largest in segmental-type (AUC difference 0.087; *p* = 0.002).

According to the recent meta-analysis by Tetreault et al. [18], only three studies have investigated the diagnostic performance of OPLL in plain radiographs. However, these studies did not include patients without target conditions as a control group and merely reported percent agreement as a measure of diagnostic performance. In a study by Mizuno et al., lateral plain radiography revealed 15 (88.2%) of the 17 OPLL cases, and the two missed cases were the segmental-type. In contrast, Kang et al. reported that the diagnostic accuracy using plain lateral radiographs was only 52.2% [4]. Furthermore, Jeon et al., in their analysis of 146 Korean OPLL patients, showed that OPLL was overlooked in 29 patients (19.9%) on plain radiography [19]. The low diagnostic accuracy of plain radiography was because the plain radiograph is a projection image, and the overlap of the lesion with the facet joint and pedicle shadow interferes with the detection of the OPLL. In addition, the ossification of PLL in segmental- and circumscribed-types can be mistaken for the posterior cortex of the vertebral body and disc calcification.

Our study demonstrated that both the DL model and radiologist performed better in continuous- and mixed-types than in segmental and circumscribed-types. This result is consistent with the findings of the previous study that the diagnostic accuracies using lateral radiographs were higher in the continuous (85.7%) and mixed-type (91.7%) than those of the segmental-type (27.3%) and circumscribed-type (20.0%) [4]. Otake et al. measured the maximum thickness of the ossified lesions on conventional lateral tomograms, and the mean thickness of segmental-type OPLL was 4.3 mm, which was smaller than those of continuous-type (9.1 mm) and mixed-type (8.2 mm) [20]. The decrease in the diagnostic performance in the segmental-type may be due to the relatively small thickness of the ossification lesions compared to the continuous-type. Moreover, ossified lesions leading from the vertebral body to the adjacent intervertebral discs, as in the continuous-type, may be more evident for radiologists to recognize as abnormalities.

A recent study by Miura et al. [8] evaluated the convolutional neural network’s (CNN) performance in diagnosing OPLL on cervical radiographs. They reported that the CNN’s accuracy, average recall, and average precision were 0.86, 0.86, and 0.87, respectively, similar to our study with the patient-level sensitivity of 91%. Also, the performance of the CNN model was higher in the continuous and mixed types than in the segmental and circumscribed types, which is consistent with our results. However, in contrast to their classification model, our study was based on the direct segmentation of OPLL lesions which may enhance the explainability and interpretability of the model.

In our study, the diagnostic performance was highest at C2 and tended to decrease as the level decreased to C7. The main reason is that the lower cervical spine can be obscured in the lateral plain radiograph in patients with a short neck and substantially elevated shoulders [21]. Therefore, it is essential to ensure that the lower cervical spine is adequately visualized by inferior traction of the arms with full expiration [22].

With the rapid development of artificial intelligence and deep learning in recent years, many researchers are actively utilizing this new technology in the field of spine imaging [23]. These studies range from the identification of vertebral fractures [24] to a diagnosis of osteoporosis [25, 26], detection of spinal bone metastases [27], and automatic measurement of spine alignment [28, 29]. With direct access to the pixel-level values and exhaustive search of the entire image, DL algorithms can automatically discover and extract sophisticated features and use them for lesion classification. However, the DL model also has limitations. When there is a lack of training data for a specific disease pattern, some straightforward tasks for radiologists can be rather difficult for the algorithm. In this study, the DL model showed higher performance than the radiologist in the segmental-type, while radiologists showed higher performance in the continuous-type. These complementary strengths of the DL model and human observer may explain the improvement of diagnostic performance when the DL model is used as a second reader.

This investigation had several limitations. First, although the diagnostic performance of the DL model alone was higher than that of the average observers, the difference was not significant. In this study, the size of the training dataset was small, and large-scale data are required to improve the performance of the DL model. Additionally, the statistical power needs to be increased through a larger number of observers and cases. Second, although we used CT images as a reference standard to manually segment the OPLL lesions on a plain radiograph, the boundary of small lesions was not clearly demonstrated on radiographs and could only be detected on CT. This inaccuracy in preparing training data would have influenced the performance of the DL model. Third, we only validated model performance using the data of the same institution as the development dataset; hence, the performance of the model may be overfitting. It is necessary to recruit sufficient OPLL cases at various institutions and perform external validation to evaluate model performance more accurately by future multicenter studies. Finally, we did not measure the change in reading time according to the use of the DL model. Several recent studies [30] have reported a reduced reading time as a strength of the DL model in clinical practice, and a follow-up study is needed to measure the change in reading time of cervical radiographs.

In conclusion, the deep learning-based OPLL detection model can significantly improve the diagnostic performance of radiologists on lateral cervical radiographs. Particularly, the DL model can help diagnose segmental-type OPLL, which shows a low diagnostic performance for radiologists.

## Supporting information

S1 Table Interobserver agreements between human observers. (DOCX)

S1 File (ZIP)
