# Supplementary material for: Improved diagnostic performance of plain radiography for cervical ossification of the posterior longitudinal ligament using deep learning
Source: PLoS One. 2022 Apr 27;17(4):e0267643. doi: 10.1371/journal.pone.0267643 (PMC9045646; doi:10.1371/journal.pone.0267643)
Supplement: S1 Table — (DOCX) [file pone.0267643.s001.docx]

**S1 Table. Interobserver agreements between human observers.**

|  | Resident 1 | Resident 2 | Fellow 1 | Fellow 2 | Staff 1 | Staff 2 |
| --- | --- | --- | --- | --- | --- | --- |
| Resident 1 | - | 0.30 | 0.35 | 0.28 | 0.42 | 0.35 |
| Resident 2 | - | - | 0.58 | 0.63 | 0.50 | 0.50 |
| Fellow 1 | - | - | - | 0.66 | 0.53 | 0.50 |
| Fellow 2 | - | - | - | - | 0.51 | 0.55 |
| Staff 1 | - | - | - | - | - | 0.56 |
| Staff 2 | - | - | - | - | - | - |
